# Self-Assembling Protein Nanoparticles in the Design of Vaccines: 2022 Update

**DOI:** 10.3390/vaccines10091447

**Published:** 2022-09-02

**Authors:** Sergio Morales-Hernández, Nerea Ugidos-Damboriena, Jacinto López-Sagaseta

**Affiliations:** 1 Unit of Protein Crystallography and Structural Immunology, Navarrabiomed-Public University of Navarra (UPNA), 31008 Pamplona, Spain; 2 Navarra University Hospital, 31008 Pamplona, Spain

**Keywords:** self-assembling protein nanoparticles, SAPNs, VLPs, virus-like particles, human papillomavirus (HPV), norovirus (NoV), hand, foot and mouth disease (HFMD), nanoparticles, immunogen, antigen, vaccine, virus, antibodies

## Abstract

Vaccines constitute a pillar in the prevention of infectious diseases. The unprecedented emergence of novel immunization strategies due to the COVID-19 pandemic has again positioned vaccination as a pivotal measure to protect humankind and reduce the clinical impact and socioeconomic burden worldwide. Vaccination pursues the ultimate goal of eliciting a protective response in immunized individuals. To achieve this, immunogens must be efficiently delivered to prime the immune system and produce robust protection. Given their safety, immunogenicity, and flexibility to display varied and native epitopes, self-assembling protein nanoparticles represent one of the most promising immunogen delivery platforms. Currently marketed vaccines against the human papillomavirus, for instance, illustrate the potential of these nanoassemblies. This review is intended to provide novelties, since 2015, on the ground of vaccine design and self-assembling protein nanoparticles, as well as a comparison with the current emergence of mRNA-based vaccines.

## 1. Introduction

The origin of vaccines dates back more than 200 years to when the first vaccine against smallpox was developed by Edward Jenner (1798). Since then, vaccines have come to represent an essential preventive tool for public health. Thanks to vaccines, several epidemics have remained under control and the devastating consequences often associated with the pathogens that surround us have been greatly diminished. Among the wide variety of vaccines that have been produced to date, many contain live-attenuated or inactivated viruses, the administration of which to a healthy individual is intended to provoke a protective response mirrored in a robust immunological memory signature [1,2]. The former is associated with potent immune responses, however, the risk of both reversion into active, pathogenic viruses as well as mutations, makes this vaccination format not the safest alternative [3]. Killed viruses, on the other hand, are unable to produce an infection in immunized subjects. However, their potency to elicit a robust immune reaction is reduced.

**Figure 1:**
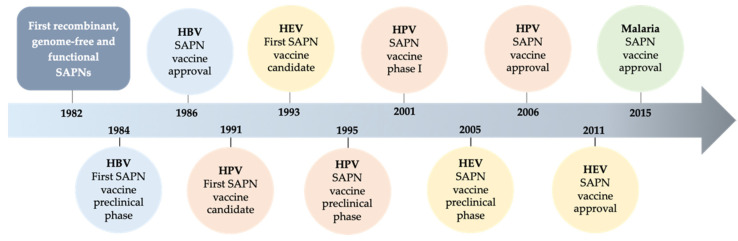
Chronology of vaccine development in the context of self-assembling protein nanoparticles. The milestones achieved by vaccinologists since 1982 are indicated. Figure created in BioRender. Abbreviations: hepatitis B virus (HBV), human papillomavirus (HPV) and hepatitis E virus (HEV).

A more recent generation of immunogen-delivery platforms surfaced some decades ago (Figure 1) and since then they contribute to counteracting these drawbacks. They do so by engaging protein subunits with the capacity to self-assemble, in the absence of any genetic cargo, into virus-like particles (VLPs), thus eliminating any potential risk derived from replication or mutation events within the host [4]. These macromolecular designs, known as self-assembling protein nanoparticles (SAPNs), contain recombinant proteins (often mimicking viral proteins), accessible to being “seen” by the immune system and initiating the desired protective immune reaction [5]. 

**Figure 2:**
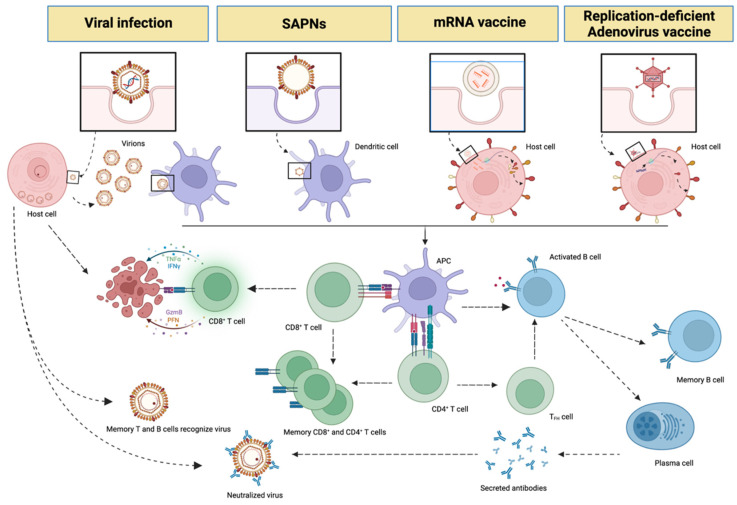
Viral- and vaccine-elicited immune reactions. The figure shows a schematic comparison of the differences and similarities regarding the elicitation of an immune response by live viruses, SAPNs, or DNA/RNA vaccines. Host immune reactions follow common pathways, regardless of whether the trigger is a natural pathogen or vaccine formulation. The main difference among these pathways is that in vaccine-elicited reactions antigen that is delivered to the host, as in the case of recombinant proteins or SAPNs, directly initiates an immune response by activating antigen-presenting cells (APCs). On the contrary, mRNA- or adenovirus-based vaccines require a prior step whereby host target cells will produce the desired immunogen. APCs fragment the immunogen into smaller peptides and present them on their surface-by-surface receptors to several types of cells in the host, such as cytotoxic (CD8^+^) T, helper (CD4^+^) T and B cells. B cells are activated by immunogen recognition, differentiate into plasma cells, and secrete antibodies that neutralize the virus. The activation of CD4^+^ T cells by APCs causes them to differentiate into different subtypes, such as T follicular helper cells (T_FH_), which also help B cells to differentiate into memory B cells and antibody-secreting plasma cells and promote the production of high-affinity antibodies. Another subset of CD4^+^ T cells differentiates into memory T helper cells. Cytotoxic T cells activation by APCs interaction cause apoptosis by cytotoxic mediators release to the host cells that are infected with the virus. Some CD8^+^ T cells differentiate into memory cytotoxic T cells, which show a fast response against secondary immunogen contact. Figure created in BioRender. PFN: perforin; GzmB: Granzyme B; IFNγ: Interferon γ; TNFα: Tumor necrosis factor α.

On the other hand, DNA- or RNA-containing vaccines are meant to deliver immunogen-coding genes or fragments thereof into the host cell using either DNA plasmids or RNA-lipid particles as vectors. These trigger the host cell to express the desired immunogens that ultimately lead to humoral and cellular immune responses dependent on antigen-presenting cells and major histocompatibility complex cell receptors [6,7] (Figure 2).

Viruses are naturally able to replicate in the host, with the aim of producing new viral progeny and perpetuating (Figure 2). The information required to complete their reproductive cycle is encoded in their genome and thanks to this, cells infected by the virus can produce all the necessary structures to assemble new virions identical to their predecessor; such as capsid proteins, which are indispensable for locating the genome, stabilising it and protecting it. These proteins are organised into capsomers, which assemble into repetitive scaffolds leaving a watertight compartment where the viral genome is located.

This idea led to the development of VLPs, another example of vaccines that could be referred to as artificial virus-like nanostructures. Their shape and composition are variable as they can be composed of all or some of the proteins of the virus capsid. A key difference lies in the lack of genetic (viral) material, which prevents the usual risks associated with vaccines using, for example, inactivated viruses [8]. Therefore, VLPs cannot replicate within the host cell but can carry viral recognition proteins or subunits and thus stimulate the immune system, generating a potent immune response through recognition of these subunits (Figure 2). Due to the advantages when compared to other types of vaccines, interest in VLP technology has increased in recent years.

The first data on recombinant and functional SAPNs dates from 1982, when Valenzuela and colleagues report the preparation of hepatitis B virus (HBV) SAPNs produced in yeast [9]. Since then, SAPNs vaccine candidates have evolved in leaps and bounds. After this achievement, the amount of new SAPN-based vaccines designed and approved for different types of pathogens has increased considerably [10], improving existing vaccines against specific viruses. Here, we update the status of this technology from the years after 2015 to the present day (Table 1).

## 2. Self-Assembling Protein Nanoparticles as Vaccine Immunogens

**Figure 3:**
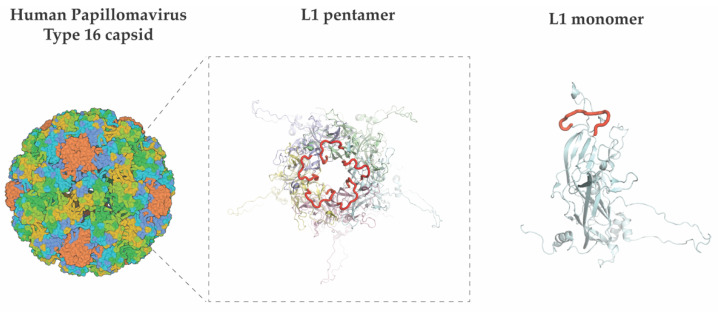
Structure and location of the DE loop in the HPV SAPN particle. The capsid of the type 16 HPV is shown in the left panel, as a surface-coloured representation. The pentamer formed by the L1 protein is displayed in a zoomed-in image in the centre panel, with each of the L1 monomers shown in individual colours in cartoon mode. The DE loop is highlighted in red colour. The right panel shows an individual L1 protein (monomer).

SAPNs are macromolecular designs with an architecture similar to that of viral capsids. Their structure is defined by replicates of protein subunits that self-assemble in a highly ordered manner to form concrete three-dimensional nanostructures. SAPNs vary in size up to about 200 nm [11], therefore, their dimensions are in the range of those of viral particles. One of the most relevant features of SAPNs is their accuracy in mimicking the outermost viral protein shells, and consequently, their immunogenicity [12] (Figure 2 and Figure 3).

SAPNs can be composed of one or several structural proteins—as found in viral particles—that bear the inherent ability to self-assemble when recombinantly expressed. Depending on the number of these structural proteins, SAPNs can be simple, as in the case of those developed to protect against Human Papillomavirus (HPV), where the SAPNs capsid consists of a single protein [13], or complex, such as those belonging to the *Reoviridae* family that contain 2–4 different proteins arranged in multiple layers [14]. 

Similar to some viruses, some SAPNs may carry an additional component, an outer lipid envelope surrounding the core. It is usually acquired by gemmation, when the capsid is enveloped in the cell membrane, as is the case of the human immunodeficiency virus type 1 VLPs. When producing SAPNs recombinantly, the cell line of choice is therefore relevant as enveloped SAPNs will present proteins found in the cell membrane [14,15]. Therefore, the design of SAPNs is not restricted to protein components, and it shows flexibility to conceptualize multicomponent nanoparticles that could be of interest in the development of novel vaccine candidates against pathogens with complex capsids.

**Table 1:** Update of SAPN-based vaccines from 2015.

Pathogen	Platform	Surface Proteins	Immunogens	Adjuvant	Disease	Model	Year	Ref.
Human papillomavirus	HPV-16/18 L1	L1	L1 y L2	AS04	Papillomatosis, oropharyngeal and anogenital cancer	Mice and rabbit	2016	[16]
	HPV-16L1/58L2	L1	L1 y L2	Alumn-MPL	Papillomatosis, anogenital and oropharyngeal cancer	Mice and rabbit	2017	[17]
	HPV-16L1	L1	L1		Papillomatosis, anogenital and oropharyngeal cancer		2016	[18]
	HPV-16L1	L1	L1		Papillomatosis, anogenital and oropharyngeal cancer	Kunming mice	2016	[19]
Norovirus/enterovirus/rotavirus	NoV- EV-RV	VP0, VP1, VP3/ VP1/ VP6	VP0, VP1, VP3/ VP1/ VP6	rVP6	Childhood gastroenteritis	Mice	2019	[20]
Norovirus	NoV-VLP	VP1	VP1		Severe acute gastroenteritis and diarrhoea	Mice and rats	2020	[21]
	NoV-VLP	VP1	VP1	Addavax	Severe acute gastroenteritis and diarrhoea	Mice and rats	2021	[22]
	P-24 nanoparticle S60-nanoparticle	VP1	VP1		Severe acute gastroenteritis and diarrhoea	Mice and rats	2019	[23]
Enterovirus 71/ Coxsackie A16/ varicella-zoster	HBc-V/1/2	HBsAg	VP1/VP2/ VZV-gE	Alumn adjuvant	HFMD/chicken pox	Mice and rats	2017	[24]
Enterovirus 71	EV-71 VLP’s	VP0, VP1 and VP3			HFMD	Mice and rats	2018	[25]
	EV-71 VLP’s	VP0, VP1 and VP3		Alhydrogel	HFMD	Mice and rats	2020	[26]
	EV-71 VLP’s	VP0, VP1 and VP3		Alumn adjuvant	HFMD	Mice and rats	2021	[27]
MERS	MERS-CoV VLP’s	M, E and S	RBD	Alumn adjuvant	MERS	Macaques	2017	[28]
SARS-CoV2	HCoV-NL63	M, E and S	RBD		COVID	Cell culture		[29]
	SARS-CoV-2 VLP’s	S, M, E and N	RBD		COVID	Mice	2020	[30]
	SARS-CoV-2 VLP’s	S, M, E and N	RBD	ODN K3-CpG + Al	COVID	Mice	2021	[31]
	CuMVTT-RBM	CuMVTT	RBD		COVID	Mice and rabbits	2021	[32]
	i301	S	RBD	AddaVax	COVID	Mice and macaques	2022	[33,34]
Epstein-barr	gH/gL-EBNA1/gB-LMP2	gH/gL/gB	EBNA1/LMP2		Mononucleosis/multiple cancers	Mice	2016	[35]
*Plasmodium*	Rv21	CSP/TRAP	PvCSP/PvTRAP		Malaria	Mice	2018	[36]
*Plasmodium falciparum*	M-HBsAgS-N4	HBsAgS	CSP		Malaria	Mice	2019	[37]
*Lutzomyia longipalpis* (Leishmania)	Influenza virusomas	LJL143/Leish3/KMP11	KMP11 and LeishF3+	GLA-SE and TLR4 agonist	Human visceral leishmaniasis	Mice	2017	[38]
*Staphylococcus aureus*	LND-VLP	AP-205	LND		Skin soft tissue infection/pneumonia	BALB/c mice	2020	[39]

Wild-type HBV capsids are composed of arrays of a protein subunit called Hepatitis B surface antigen (HBsAg), which is the major capsid protein. Its distribution and multiplicity in the three dimensions produce a spheric pattern of particles of around 42 nm in size. This is an important feature as a size range in the 20–200 nm range allows SAPNs to enter the lymphatic vessels [40].

Many of the new vaccines based on SAPNs make use of HBV scaffold proteins like HbsAg due to their small size and their structural adaptability to display varied immunogens that can be mounted on the particle surface to elicit an immune reaction. Therefore, it is possible to create protein fusions leading to chimeric SAPNs where the core, self-assembling protein belongs to one type of virus, whilst the immunogen derives from another pathogen against which a protective immune response is intended. 

Therefore, by fusing both core and immunogenic proteins, one can achieve novel SAPNs with the immunogen expressed (*i*) at a great density, (*ii*) in a highly ordered manner, (*iii*) preserving its native conformation, and (*iv*) accessible for recognition of key target epitopes by immune elements such as antibodies and B-cell receptors (BCRs) that could neutralize the pathogen in a potential event of a natural infection. 

As mentioned earlier, and as it occurs in nature with real viruses, where multiple proteins are displayed on the surface of the virus capsid, a variety of immunogens can be presented on a common scaffold at a high density. Such designs strengthen the avidity established between the immunogens and the BCRs. This scenario promotes clustering and uptake by immune cells that favour an enhanced immune response [41,42]. Nonetheless, the nature of the shell architecture might associate with structural and steric restrictions that need to be taken into account in the design of recombinant SAPNs for multi-epitope presentation on a single scaffold. The immune system is provided with a further mechanism for the recognition of pathogens which involves molecular sensors for pathogen-associated molecular pattern molecules, widely known as PAMPs. The recognition of these motifs is driven by proteins such as Toll and Nod-like receptors, which constitute two major components of the host’s innate immune system [43]. Given their ability to recognize foreign PAMPs, these receptors contribute to the robust immune stimulation triggered by SAPNs, which have the important property of preserving the native state of foreign molecular motifs.

In vaccinology, the advantages conferred by SAPNs have been already harnessed to develop several vaccine candidates to protect against varied infectious diseases. Perhaps the most outstanding examples are marketed SAPNs vaccines conceived against HBV, such as Recombivax HB^®^ (Merck, Rahway, NY, USA), Engerix-B^®^ (GlaxoSmithKline, Brentford, United Kingdom), Elovac B^®^ (Human Biologicals Institute, Kozipannai, India), Genevac B^®^ (Serum Institute, Hadapsar, India), and Shanvac B^®^ (Shantha Biotechnics, Hyderabad, India). Another relevant example is provided by SAPNs vaccines to protect against HPV. These constitute the main component of Gardasil^®^ (Merck) and Cervarix^®^ (GlaxoSmithKline). One further example is found in Hecolin^®^ (Innovax, Xiamen, China), a recombinant vaccine developed against the Hepatitis E virus.

### 2.1. Evolution of SAPNs and Vaccines

Throughout history, vaccines have helped humankind fight and prevent diseases caused by pathogens of different natures, such as viruses, bacteria, or even parasites; some of which are the cause of serious diseases.

#### 2.1.1. Human Papillomavirus (HPV)

HPV is an uncoated double-stranded DNA virus and the main aetiological agent of cervical cancer and anogenital preneoplastic lesions [44]. The SAPNs initially designed against HPV are based on the L1 protein, a key component of the viral capsid architecture [45]. The L1 protein self-assembles when expressed recombinantly in varied expression systems such as yeast or insect cells [46]. Gardasil^®^ and Cervarix^®^ are two well-known marketed HPV vaccines constituted by SAPNs. Both vaccines protect against HPV types 16 and 18, which are cancer-associated serotypes. However, each has a different production strategy, as these are produced in *Saccharomyces cerevisiae* and *Trichoplusia ni* insect cells, respectively [46].

It would be desirable to achieve a vaccine against HPV with broad amplitude and high capacity to activate the immune response. Chimeric SAPNs represent a powerful tool, as different antigen epitopes can be mounted on a single particle and elicit a more robust protective response against heterogeneous epitopes. This strategy has been assayed to improve the immune response of HPV vaccines. Several chimeric HPV SAPNs have been recombinantly generated by grafting L2 minor capsid protein epitopes into the DE loop or the C-terminus of L1 (Figure 3). 

Some of these chimaeras have been produced by inserting the regions defined by (*i*) amino acids 17 to 36 of the L2 protein of HPV-33 and (*ii*) amino acids 56 to 75 of the L2 protein of HPV-58, into the DE loop of the L1 protein of HPV-18. In combination with Adjuvant System 04™, the best results were obtained in experimental animal models by grafting L2 on L1 of HPV-16/18. In mice and rabbit animal models, these formulations induced persistent immune responses and protection against different HPVs [16].

Unlike the three currently licensed SAPN vaccines, which are constituted by the L1 major capsid protein of up to four or nine different HPV serotypes [47], this vaccine contains SAPNs based on a recombinant chimaera of two highly conserved neutralising epitopes of the L2 minor capsid protein grafted on L1.

Chen et al. developed a new HPV SAPN chimaera whereby amino acids 16–37 of the HPV serotype 58 L2 minor protein were grafted on the DE loop of HPV-16 L1 major capsid protein [17]. Together with aluminium monophosphoryl lipid A as an adjuvant, this formulation yielded good results for a broad group of HPV serotypes in murine and rabbit models. This chimeric SAPNs is, therefore, a candidate to be further considered.

Other approaches related to vaccines based on SAPNs are being explored. Two strategies are focused on improving recombinant protein yields by using alternative expression systems or genetic modifications in the protein-expressing cells. An illustrative example is provided by the production of SAPNs in *Escherichia coli* as an expression system for HPV-16 L1 protein. By using codon-optimized genes and a bioreactor system, Bang et al. achieved high protein yields (4.6 g/L) in the *E. coli* BL21DE3 strain [18]. On the other hand, Chen et al. [19] observed that fusion of the HPV-16 L1 target protein with the Small Ubiquitin-like Modifier (SUMO) protein, which makes the protein soluble, yielded the best results amongst a series of diverse L1 protein constructs designed for expression in *E. coli*. The proper assembly of the L1 chimeric SAPNs was confirmed via transmission electron microscopy upon removal of the tag. Mice showed a potent humoral response upon immunization with HPV-16 SAPNs. These results open new avenues for the use of new expression systems, intending to reduce production costs by obtaining safe vaccines that generate effective antibodies against HPV.

#### 2.1.2. Norovirus (NoV)

Human Noroviruses (HuNoV) belong to the calicivirus family and are causative agents of acute gastroenteritis in humans. Preventive strategies for susceptible groups such as children or the elderly have been proposed, as there are approximately 1.5 million deaths caused by HuNoV worldwide every year [48]. The search for a vaccine against norovirus (NoV) has faced diverse issues: genetic/antigenic diversity, lack of knowledge regarding the viral cycle, the absence of a permissive line for cell culture, or a successful animal model for testing the potential of vaccine candidates against this kind of virus [49].

**Figure 4:**
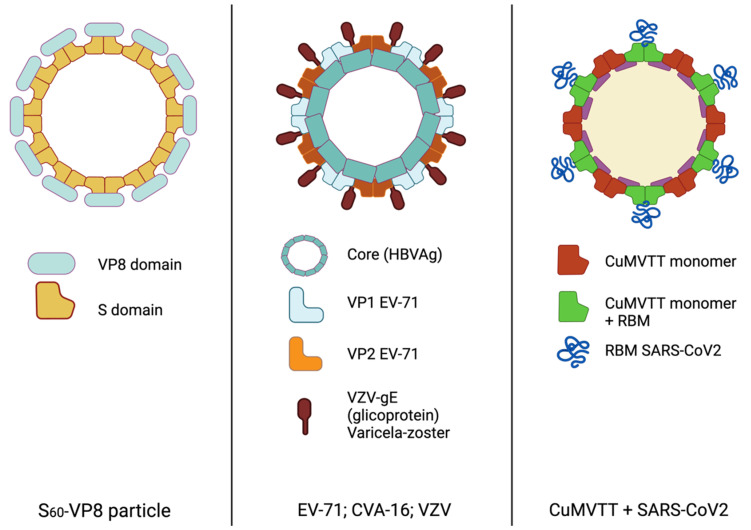
Structural flexibility in the design of SAPNs. Schematic view of three different designs of SAPNs with the core self-assembling proteins, and immunogenic epitopes mounted for surface display. Figures created in BioRender.

Structurally, HuNoV forms icosahedral particles of about 27–30 nm in diameter without envelopes [50]. Two structural proteins are the main building blocks of the viral particle [51]. The major one, VP1, is responsible for particle assembly and contains two domains, S and P. The former makes up the viral shell, while the latter forms the protrusions of the viral shell (Figure 4) that bind the host receptor histo-blood group antigens. On the other hand, VP2 is a structural minor capsid protein [52,53].

With regard to NoV, other types of nanoparticles known as P-particles have been developed. Their name comes from the protein domain VP1 found in the viral capsid. This domain is exposed outwardly, and it is known as P (protruding) [54]. In 2021, Ming and colleagues reported an engineered trivalent rotavirus (RV) nanoparticle formed by a 24-mer (P) component combined with 24 VP8 surface-exposed domains of three different RV P types. Sera of immunized mice showed high neutralization activity against replication of three RV types, paving the way for the design of novel and efficient P RV vaccine candidates [55]. A similar yet larger type of efficient P RV pseudoparticles [56] is also being conceived in a trivalent format [57].

Current vaccine candidates are based on the inoculation of SAPNs consisting of P-particles made from the protruding domain of VP1 [58], or viral vectors carrying an insert of the NoV capsid gene produced by bioengineering technologies [49]. Polyvalent vaccines have been developed to include multiple genotypes of NoV and/or other enterically acquired viruses [59]. In 2020, an innovative combination vaccine was developed to cover three of the main agents causing severe gastroenteritis in children with high mortality: RV, NoV, and Coxsackie B enterovirus (CVB). The complex formulation contained, on the one hand, NoV GII.4 VP1 SAPNs, VP0, VP1 and VP3 CVB SAPNs; and VP6 from RV, which presents a tube-like morphology in electron micrographs. Produced in insect cells and tested in BALB/c mice these SAPNs, alone or in combination, showed evidence for the generation of type 1 and 2 IgGs, indicating a mixed T helper cell type 1 (Th1)/Th2-mediated immunity response [20].

In 2012, Parra et al. [60] reported novel NoV GII.4 monovalent and bivalent SAPN formulations and in 2017, an additional bivalent SAPN vaccine against NoV genotype GI.1 and GII.4 was developed [61]. Furthermore, in order to improve both efficacy and protection, Verardi and colleagues [21] proposed the use of disulphide bonds between SAPN protomers to prevent their dissociation while promoting their stabilization. This strategy led to higher and more selective antibody titres in mice, as the surface epitopes were more accessible for molecular recognition by specific antibodies. Consequently, this macromolecular model proposes a novel candidate that associates with enhanced vaccine-mediated protection.

An important novelty introduced in the development of a VLP vaccine against NoV is the use of an alternative expression system, in this case, the protozoan *Leishmania tarentolae*. *L. tarentolae* offers an easy model for protein production with the capacity to perform posttranslational modifications, a cost-effective, safe, fast, and high-yield protein production system. Using this expression system, Panasiuk M et al. demonstrated that VLPs expressed in *L. tarentolae* elicited a potent immune response and the production of neutralizing antibodies [22].

#### 2.1.3. Hand Foot Mouth Disease (HFMD) and Varicella

HFMD affects infants, children, and adults. Symptoms of HFMD include fever, oral ulcers, vesicles on the hands and feet, and rarely even severe complications including injury to the central nervous system [62]. Further, HFMD causes a significant number of hospitalisations and deaths annually [63]. The main causative viruses are enterovirus 71 (EV71), and coxsackievirus A16 (CVA16) [64]. In EV71, proteins VP0, VP1, and VP3 make up the viral capsid. VP0 can be cleaved into two more proteins, VP2 and VP4, which are associated with infectivity [65].

Another pathogen that targets children and infants is varicella, caused by a varicella-zoster herpes virus (VZV). A chimeric single SAPNs vaccine was investigated in 2017 to prevent both HFMD and varicella. Wu et al. developed different particles with a common scaffold, the hepatitis B core antigen (HBcAg), and differently ordered surface exposed VP1 and VP2 epitopes from EV71, and gE from VZV (Figure 4). This design enabled the production of antiserum that turned out to cross-neutralise CVA16, which also triggers HFMD, in BALB/c mice. This candidate is at a pre-clinical stage and represents a promising vaccine development [24].

Several advances have been achieved in subsequent years concerning vaccine production against EV71. For instance, co-expression of EV71 P1 and the 3CD protease allows cleavage of the P1 protein, leading to VP0, VP1, and VP3 subunits and the formation of the particle shield [25]. Here, Kim HJ. Et al. introduced several promoters such as the immediately early full-length cytomegalovirus promoter (CMV-IE), lef3, gp41, and chitinase promoters. This vaccine candidate was tested in vivo, leading to neutralizing anti-EV71 Ig in mice.

The optimization of recombinant protein expression systems for high throughput production is important from a cost-effectiveness point of view. In this line, Zhijian Yang and colleagues produced P1 and 3CD proteins from EV71 in *Pichia pastoris*, a widely used yeast cell line for recombinant protein expression. The use of aluminium-containing adjuvants improved the response in mice, achieving a high serum titre [26]. It is therefore a candidate vaccine for the prevention of HMFD.

In 2021, another SAPNs-type vaccine against EV71 was produced in *P. pastoris*, and tested in mice and non-human primates, evaluating immune response and toxicity [27]. Biochemical and biophysical analyses determined that the ~35 nm spherical SAPNs consisted of processed VP0, VP1, and VP3 proteins and that adding aluminium-based adjuvants provided effective protection in vaccinated mice, as the antisera showed cross-neutralisation activity against EV71. Moreover, no signs of systemic toxicity were found in the animals tested. Based on the data provided, this vaccine could be a candidate for further clinical trials.

#### 2.1.4. Severe Acute Respiratory Syndrome-Related Coronavirus (SARS-CoV)

In the most recent years, the emergence of the COVID-19 pandemic swept along a blast of vaccine candidates against SARS-CoV-2 [66,67]. Other related beta coronaviruses (CoV) such as SARS-CoV [68,69] and the Middle East respiratory syndrome-related coronavirus (MERS-CoV) [70] caused previous outbreaks in 2003 and 2012, respectively. Since then, diverse strategies have been undertaken in the prevention of SARS, particularly vaccines based on SAPNs [71]. CoVs are enveloped RNA viruses with high transmission rates and the potential to cause severe or fatal illnesses [66]. The viral shell of CoV is composed of four proteins: (1) the nucleocapsid (NP) binds to the RNA genome and enables the constitution of the nucleocapsid; (2) the spike protein, often referred to as S protein, is essential for colonization of the host by engaging the host receptor ACE2; (3) the membrane (M); and (4) envelope (E) proteins which contribute to the overall organization and shape of the viral capsid. The outer lipid envelope provides these viruses with a characteristic crown shape under the microscope, due in part to the heavy glycosylation moiety present on the surface of the virus.

In 2017, Wang C. et al. [28] generated SAPN vaccine candidates against MERS. The candidate was composed of S, E, and M recombinant proteins produced using baculovirus and insect cells as the system for the expression of these components. In animal experimental models, the immune response using these SAPNs produced not only a response characterized by specific virus-neutralising IgG antibodies against the receptor binding domain (RBD) of the S protein but also cellular immunity in a Th1 cell-dependent manner.

Other SAPNs against CoV were reported in 2018, where structural proteins M, E, and S were co-expressed in a baculovirus expression system. By evaluating its ability to self-assemble and transduce responses in cells expressing the ACE2 receptor [29], the presence of this receptor was shown to be a prerequisite for the internalisation of the particle.

Ruodan Xu and colleagues [30] demonstrated that both the M and E proteins are necessary for viable confirmation and release of SARS-CoV-2 SAPNs. They also showed that production in Vero E6 cells leads to more stable SAPNs compared to those produced in HEK-293T. Using VLPs, Yilmaz and co-workers were able to confer protection against COVID-19 upon immunization with VLPs containing all four structural SARS-CoV-2 antigens [31].

But certainly, one of the most striking advances is the introduction of the genetically engineered SARS-CoV-2 receptor binding motif (RBM) on the surface of the immunologically optimised cucumber mosaic virus (Figure 4). In contrast to previous proposals for expression, a bacterial system was used for the production of this chimeric protein, which multiplied the yield via large-scale production. Furthermore, their results are optimistic as robust immune responses were elicited in both mice and rabbits, inducing neutralizing, long-lasting antibodies of higher avidity compared to convalescent human sera [32].

Following a previous design of a 60-mer SAPN derived from the hyperthermophilic bacterium *Thermotoga maritime* [34], Cohen A. et al. reported recently mosaic nanoparticles conceived to display the SARS-CoV-2 plus seven additional SARS-like CoV spike RBDs (mosaic-8) [33]. Compared to nanoparticles with only the SARS-CoV-2 spike RBD attached, which afforded protection to only the SARS-CoV-2 challenge, immunization of mice and macaques with mosaic-8 led to a broad array of cross-reactive antibodies and protection against SARS-CoV-2 and SARS-CoV-1.

#### 2.1.5. Epstein Barr Virus (EBV)

EBV causes infectious mononucleosis and is associated with malignant tumours such as nasopharyngeal carcinoma, gastric carcinoma, and multiple lymphomas (Hodgkin’s, Burkitt’s), as well as multiple sclerosis [72].

The surface of EBV is coated with diverse glycoproteins (gp) that are produced in new virions by sequestering the cellular machinery of infected cells. These gps have been used in vaccines that are currently under development. By engaging CD21 and CD35 receptors, gp350/220 are key for the interaction of EBV with B cells. Following this binding, the virus is taken up via fusion of the viral and host cell envelopes through an endocytic process mediated by the interaction between EBV gp42 and MHC class II molecules.

In an innovative study, Perez EM et al. assembled two EBV proteins that have not been used before in clinical trials. These are the latent membrane proteins EBV nuclear antigen 1 (EBNA1) and the latent membrane protein 2 (LMP2). Chimeric and polyvalent SAPNs were produced in Chinese hamster ovary (CHO) cells, which are associated with high titres of neutralising antibody production and T cell-specific response in mice [35].

#### 2.1.6. Malaria

Malaria is a tropical disease transmitted by the *Plasmodium falciparum* parasite through the bite of the vector *Anopheles* mosquito. It affects millions of people every year in countries of Asia and Latin America [73].

Once the parasite has been transmitted to the host, sporozoites enter the bloodstream and reach the liver where they develop, replicate, and infect blood erythrocytes. One of the main difficulties in eliminating malaria is hypnozoites, which are latent forms of sporozoites that can become active after some time [74]. Further complications related to the finding of an effective vaccine against malaria are caused by *Plasmodium vivax* due to the lack of preclinical models to test protective efficacy. Difficulties in the maintenance of *P. vivax* cultures for long periods with in vitro cultures, or access to animal models such as chimpanzees are also known drawbacks in the development of malaria vaccine candidates.

The first vaccine generated against a parasitic disease, RTS,S/AS01 (Mosquirix^®^) was approved for use in 2015 [75]. It is a SAPN vaccine that combines (*i*) a target region against which it elicits neutralizing antibodies, (*ii*) a T-cell epitope of *P. falciparum* circumsporozoite protein (CSP), and (*iii*) a platform constituted by the self-assembling HbsAg protein. Together, these proteins are expressed in the yeast *S. cerevisiae* to yield the resulting SAPN vaccine [76].

In 2017, the SAPN malaria vaccine Rv21 was developed using HBsAg loaded with *P. vivax* CSP (PvCSP) antigen [76]. Immunization studies determined that Rv21 provides protection in rodent models against malaria, where the presence of IgG was associated with protection in the absence of PvCSP-specific T-cell responses.

Then in 2018, a *P. vivax* vaccine carrying multiple antigens was designed [36]. This improvement of the Rv21 vaccine brought together the PvCSP and thrombospondin-related adhesion protein (PvTRAP) proteins on the same platform. As above, Atcheson and colleagues used a similar model, a transgenic *Plasmodium berghei* parasite that concomitantly expresses PvCSP and PvTRAP. It was tested in mice previously immunised with Rv21 and evaluated for efficacy against malaria infection both in the presence and absence of an adjuvant (AddaVax^®^ and Matrix-M^®^). The highest anti-PvCSP antibody titres were obtained in the case of Matrix-M^®^, and the mixture of both PvCSP and PvTRAP viral vectors enhanced immunity above the levels provided by individual vaccines [36].

One further improvement was later included in a malaria vaccine candidate with the addition of the amino acid sequence repeat “Asn-Ala-Asn-Pro” (NANP) of the CSP protein of *P. falciparum*, within the main antigenic site of the HBsAg. Using this strategy, SAPNs with a high density of these antigenic NANP sequences were generated, with either four or nine repeats. These chimeric SAPNs induced high anti-CSP titres in BALB/c mice, regardless of the number of NANP repeats. In contrast, the number of NANP repeats had an impact on the vaccine-induced antibody activity as measured by complement binding to CSP, one of the proposed effector mechanisms for neutralisation of *Plasmodium* parasite in vivo. Antisera from mice immunised with these SAPN vaccines containing nine NANP repeats performed better in the complement fixation assay than the group with four NANP repeats. Therefore, in this case, the immune response depends on the number of epitopes available in the SAPNs and may therefore help in future vaccine designs in general [37].

#### 2.1.7. Leishmania

Several microorganisms are responsible for leishmaniasis, such as the parasites *L. donovani* and *L. infantum*. *Leishmania* is capable of infecting phagocytes and cells of the spleen, liver, and bone marrow. It is transmitted through the saliva of female sandfly insects and causes thousands of cases and deaths per year [77,78]. Although there are medical treatments for the treatment of the disease, there is currently no vaccine available to prevent human leishmaniasis [79].

In 2017, Cecílio P et al. evaluated the immunogenicity of a SAPN vaccine candidate in vivo against human leishmaniasis [38]. The vaccine candidate contained different proteins: KMP-11 membrane protein, LeishF3 parasite protein, and LJL143, a sandfly salivary antigen. On top of this, the vaccine formulation is adjuvanted with glucopyranosyl lipid A, a TLR4 agonist. The immunogenicity conferred by this vaccine was confirmed by antigen-specific humoral and cellular responses, which were higher in immunized groups compared to controls.

#### 2.1.8. Bacteria

Several SAPN vaccines are currently under development against various bacterial infectious diseases. *Staphylococcus aureus* is one of the most frequent causes of morbidity and mortality worldwide and causes a wide range of diseases, from moderately severe skin infections to fatal pneumonia and sepsis [80]. *S. aureus* is characterised by the ability to form pores in the host cell membrane thanks to a cytotoxin, α-hemolysin, which acts as a virulence factor [81]. The cytotoxin α-hemolysin promotes infection leading to tissue damage, excessive inflammation, and lysis of innate and adaptative immune cells. Strategies to act on this pathway are therefore clinically relevant to attenuate the effects resulting from infection with *S. aureus*.

The linear neutralizing domains of Hla (LND), of about 21 amino acids long [82], are involved in Hla heptamerisation and it has been shown that an antibody against this epitope can neutralize Hla activity. Using constructs derived from the bacteriophage Qβ and AP205 (bacteriophage coat protein) Joyner and colleagues generated LND SAPNs in *E. coli* [39] Neutralization experiments showed that antibodies elicited via immunization prevented lysis of Jurkat cells in vitro and conferred protection to mice, with a reduction in both tissue damage and presence of neutrophils. In addition, these antibodies were found to bind to the native toxin and the LND peptide, suggesting high specificity.

## 3. Messenger RNA (mRNA), Non-Replicative Adenovirus Vaccines and SAPNs

Several new vaccine formats have emerged in the last few years [83]. mRNA vaccines deserve particular attention, as their use in humans began recently with the COVID-19 pandemic and has led to massive vaccine campaigns worldwide in the prevention of infection and development of SARS. These mRNA vaccines are administered in the form of nanoparticles where the RNA material is protected by an outer lipid envelope that will eventually facilitate cellular entry. Cells that uptake these nanoparticles are then genetically programmed to produce the SARS-CoV-2 spike protein on the cellular surface [84], which in turn triggers a strong immune response in the vaccinated individual. Therefore, unlike attenuated or inactivated virus vaccine approaches, mRNA vaccines do not require the introduction of whole viruses, which represents a safer vaccination approach (Table 2).

From an immunological point of view, mRNA vaccines can generate humoral but also cell-mediated immunity in vaccinated individuals [85,86]. A known drawback of these vaccines is that mRNA is not very stable and is susceptible to degradation [87].

Another type of vaccine that has been developed and approved to protect against SARS CoV-2 uses non-replicative adenoviruses. Adenoviruses are double-stranded DNA viruses genetically engineered to deliver the target gene, i.e., the antigen, into the host cells. The cells that have received the exogenous material will then express the desired antigen on the cell surface. An example of an approved vaccine using this technology is the Gam-COVID-Vax (SputnikV) vaccine [88], composed of two recombinant adenoviruses, rAd26-S and rAd5-S. The purpose of these two vectors is to administer each of them in two successive rounds of vaccination, thus stimulating the immune response in the long term. Adenovirus-based vaccines can pose a risk for immunocompromised individuals, as they can reactivate latent infections [89].

## 4. Future Perspectives

SAPNs are most frequently produced in yeast, insect, and mammalian cells or bacteria. In 2021, Kurokawa and colleagues [90] produced RV SAPNs in plants that showed good tolerance and elicitation of neutralizing antibodies in infants. Although these systems are already in use today, it is expected that the production of SAPNs in plants will become more widespread. Current candidates produced in plants include RV vaccines produced in *Nicotiana benthamiana* [90] and the quadrivalent (QVLP) vaccine [91], also produced in the same organism and which carries four haemagglutinin antigens from different strains of influenza. Indeed, this vaccine candidate has reached phase 3 clinical trials. Vaccines against SARS-CoV-2 [92] and Zika virus [93] have also been produced using these systems.

In the area of vaccine nanoparticles, amongst the most frequent alternatives aimed to potentiate the host immune response vaccinologists routinely explore, on the one hand, the use of immunogenic small protein domains rather than whole antigen proteins, and on the other hand, the production of the antigens of interest in diverse protein expression systems. With the same goal in mind, one additional element to assess is the use of novel adjuvants of higher potency, or toll-like receptor agonists [38]. Other strategies put the focus on the display of various antigens on an individual SAPN core, as well as the employment of different surface densities of such antigens [10]. Indeed, the availability of polyvalent vaccines [24] would enable vaccinologists to face multiple pathogens in a shorter period of time.

## 5. Summary

Traditional vaccines have so far provided an effective way to control many infectious diseases. However, the rapid emergence of new pathogens as recently witnessed with SARS-causing beta coronaviruses demands novel strategies to overcome the challenges that humankind might face in the future. In the search for more cost-effective, safe, and efficient vaccine candidates, researchers have tested alternative expression systems for the production of SAPNs and the concomitant use of adjuvants to stabilize the vaccine formulations and potentiate the immune responses.

SAPNs constitute a safe and very versatile type of vaccine platform due to their ability to incorporate different antigenic components on a unique platform. Their inherent ability to self-assemble enables the efficient formation of the particles in the expression system and functional surface exposure of varied epitopes. Through vaccination, SAPNs provide humoral and cellular immune responses, and consequently the production of neutralizing antibodies capable of recognizing conformational and native epitopes to repress the pathogen.

Over the years, there have been many advances in the field of SAPN vaccines that have helped researchers and industrial bodies design novel vaccine candidates that confer safe, robust, and long-lasting immune responses. Given the numerous and successful vaccine candidates conceived in the last years, SAPNs remain a powerful tool in the design of robust and safe vaccines for the upcoming years.

## Figures and Tables

**Table 2:** Comparison amongst SAPN, RNA- and DNA-based vaccines.

	SAPNs Vaccines	mRNA Vaccines	Non-Replicating Adenovirus Vaccines
**Advantages**	Safe (no genetic material) Efficient immune response Cost-effective and stable	Safe (no viral replication) Efficient inmune response Cost-effective and stable	Safe (non-replicative vectors) Efficient immune response Can be scaled up to high titer stocks
**Disadvantages**	Selected expression systems Some need adjuvants Spacial and conformational antigen limitation	Fragile No genetic errors allowed Delivery limitation	Development of resistance to specific adenoviruses No genetic errors allowed Dangerous in immunosuppressed or transplant patients
